# Supplementary figures and images for: Targeting FKBP51 prevents stress-induced preterm birth
Source: EMBO Mol Med. 2025 Mar 17;17(4):775–96. doi: 10.1038/s44321-025-00211-9 (PMC11982339; doi:10.1038/s44321-025-00211-9)

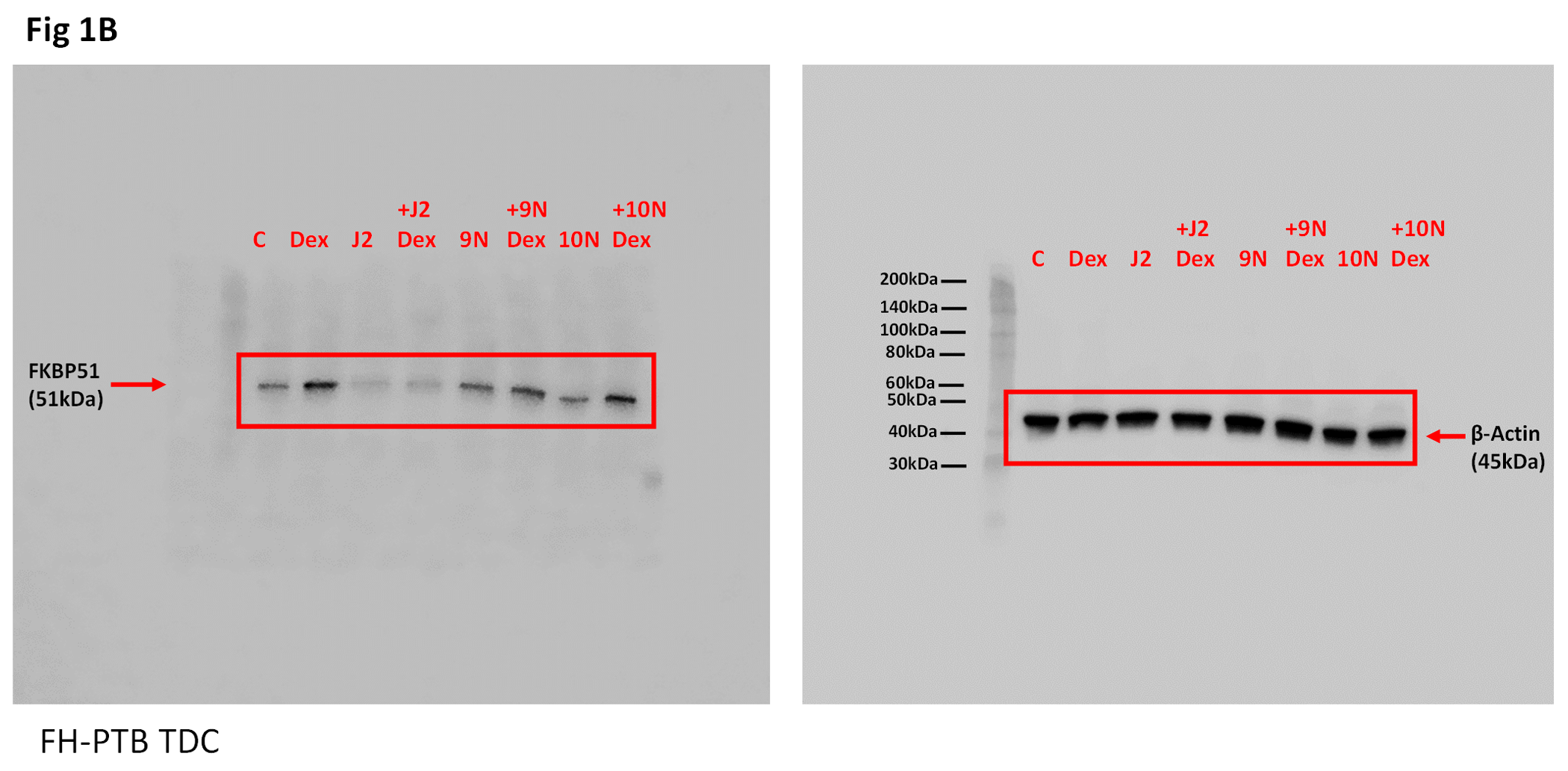

Supplement: Supplementary file 3 — Source data Fig. 1 [file 44321_2025_211_MOESM3_ESM.zip › Figure 1/Figure 1B Immunoblot.tif]

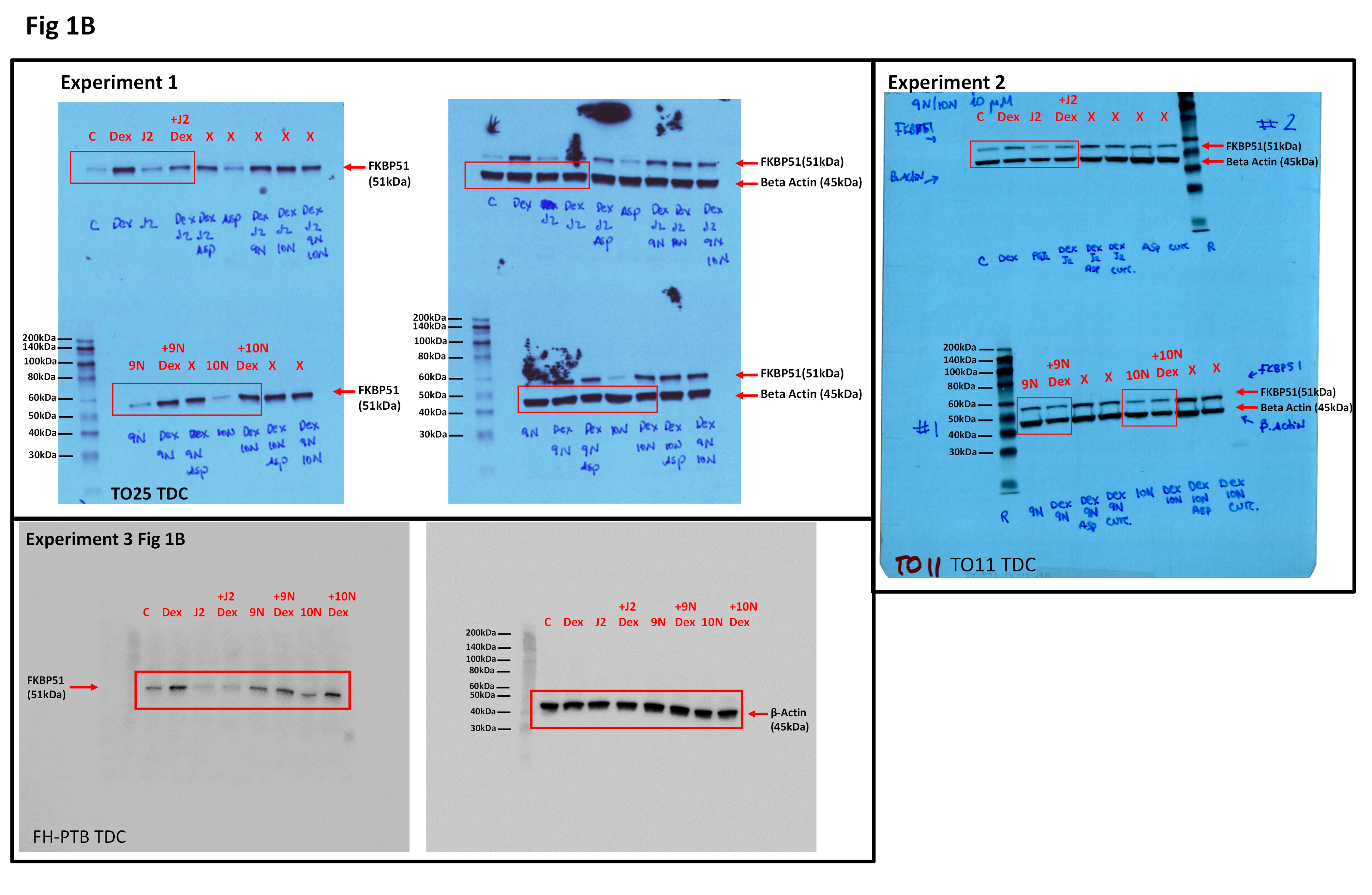

Supplement: Supplementary file 3 — Source data Fig. 1 [file 44321_2025_211_MOESM3_ESM.zip › Figure 1/Figure 1B whole immunoblots.tif]

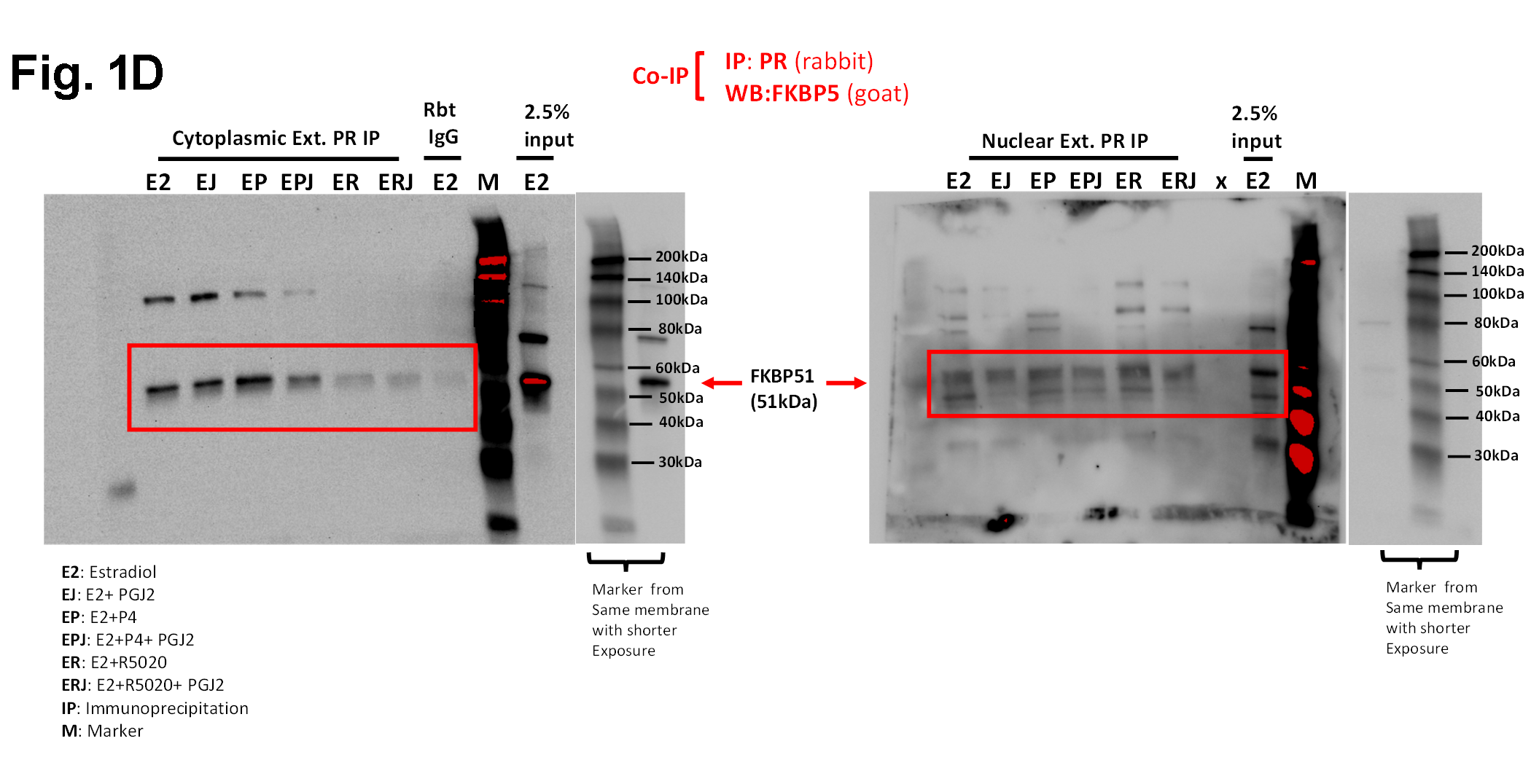

Supplement: Supplementary file 3 — Source data Fig. 1 [file 44321_2025_211_MOESM3_ESM.zip › Figure 1/Figure 1D Immunoblot FKBP51.tif]

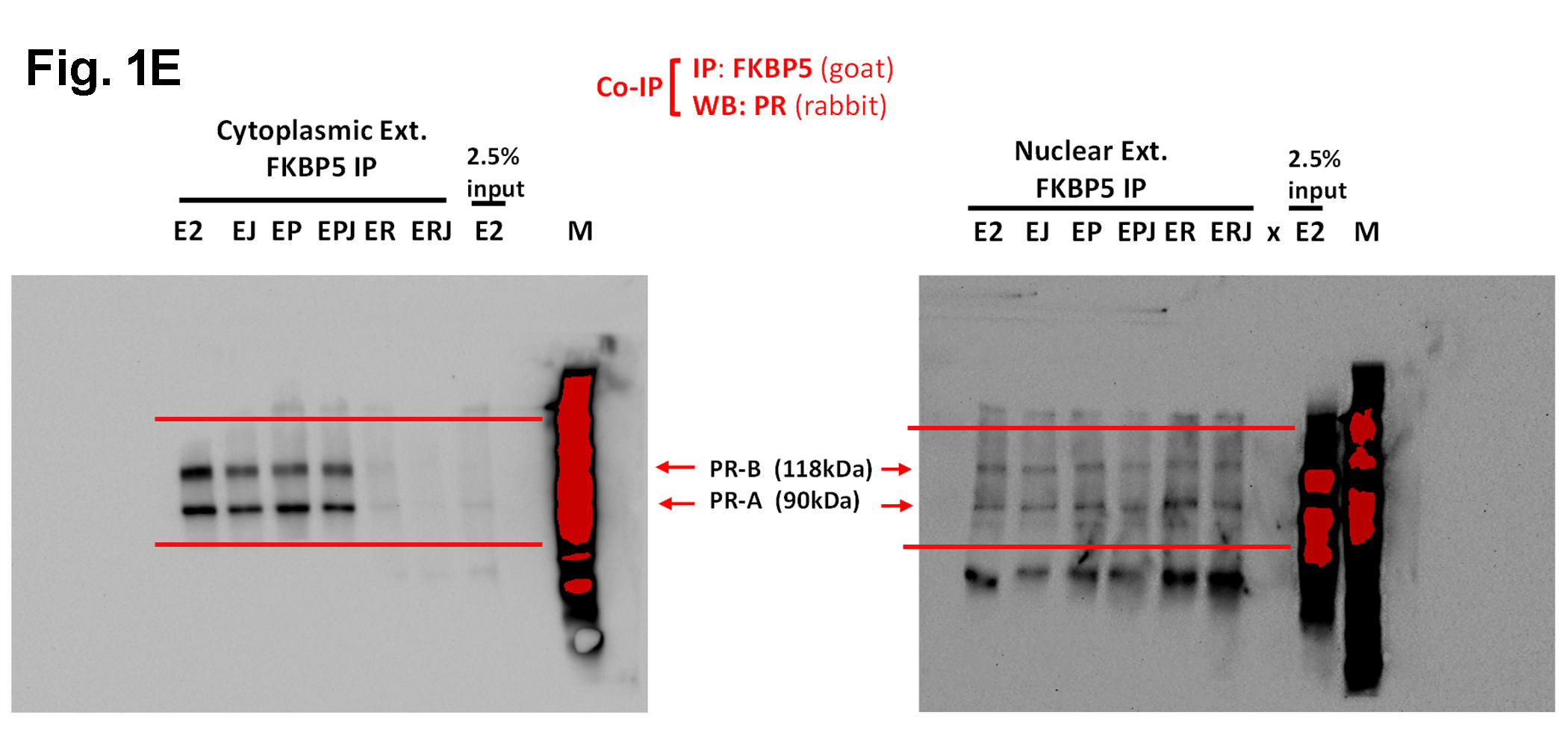

Supplement: Supplementary file 3 — Source data Fig. 1 [file 44321_2025_211_MOESM3_ESM.zip › Figure 1/Figure 1E Immunoblot PR.tif]
