# Supplementary material for: Targeting FKBP51 prevents stress-induced preterm birth
Source: EMBO Mol Med. 2025 Mar 17;17(4):775–96. doi: 10.1038/s44321-025-00211-9 (PMC11982339; doi:10.1038/s44321-025-00211-9)
Supplement: Supplementary file 9 — Expanded View Figures [file 44321_2025_211_MOESM9_ESM.pdf]

Expanded View Figures

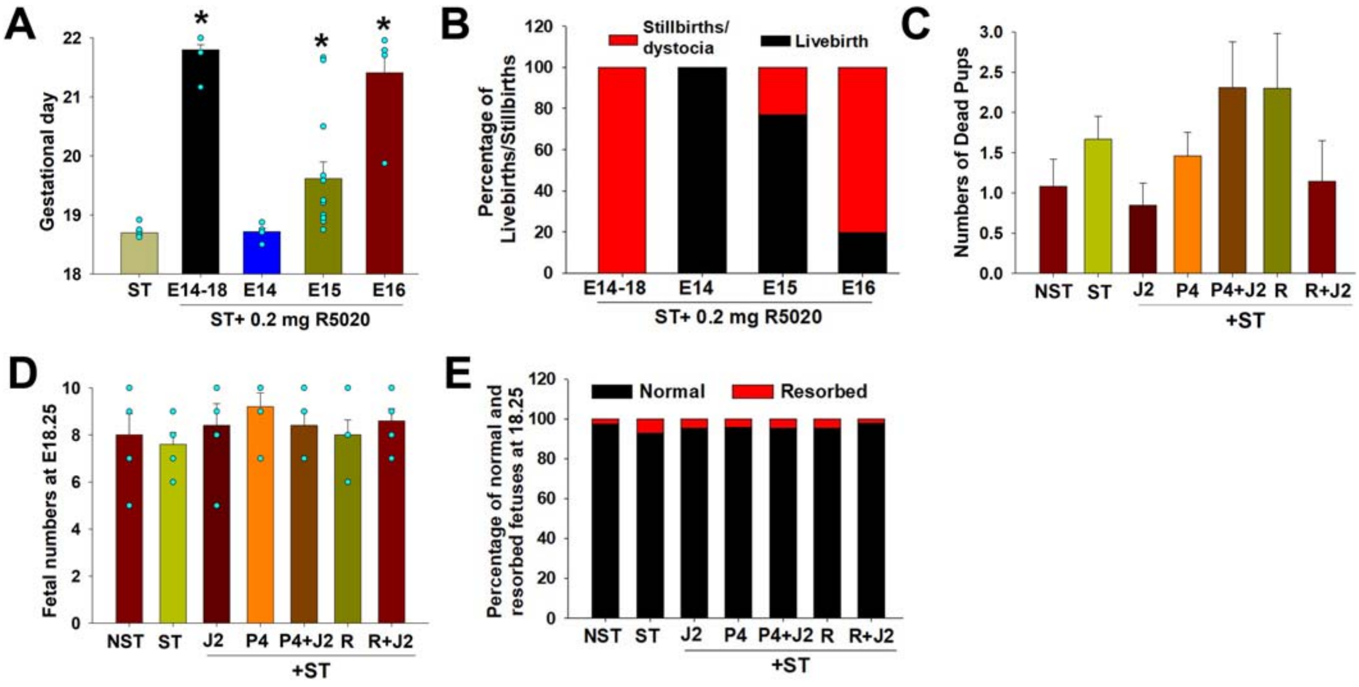

Figure EV1. Gestation day dependent effects of R5020 injections in preventing maternal stress induced PTB.

(**A**, **B**) Gestational length (**A**) and percentage of live and still births (**B**) in time-pregnant wild-type mice subjected to maternal restraint stress (ST, $n = 12$) between E8 and 18 and received s.c. injection of 0.2 mg/dam R5020 from E14 to 18 ($n = 9$), or only on E14 ($n = 5$), only on E15 ($n = 13$) or only on E16 ($n = 5$). Bars represent mean ± s.e.m. (**A**) *$P < 0.05$ vs. ST, analyzed with one-way ANOVA followed by Dunn's method. (**C**) Number of dead pups in mice left undisturbed state as control (NST, $n = 12$) or subjected to restraint stress between E8 and E18 and received from E14 to 18 injection of placebo (ST, $n = 12$) ± 15dPGJ2 (J2, $n = 13$), or P4 (P4, $n = 13$) or P4 + 15dPGJ2 (P4 + J2, $n = 13$), or R5020 (R, $n = 10$) or R5020 + 15dPGJ2 (R + J2, $n = 8$). Bars represent mean ± s.e.m. $P = 0.09$, analyzed by One-Way ANOVA. (**D**, **E**) Total fetus numbers (**D**) and percentage of normal and resorbed fetuses (**E**) at E18.25 in NST or ST or J2 or P4 or P4 + J2 or R or R + J2. Bars represent mean ± s.e.m.; $n = 5$ biological replicates. (**D**) $P = 0.7$, analyzed by One-Way ANOVA. Note that R5020 was only single injection given on E15 (**C**, **D**).

 © The Author(s)

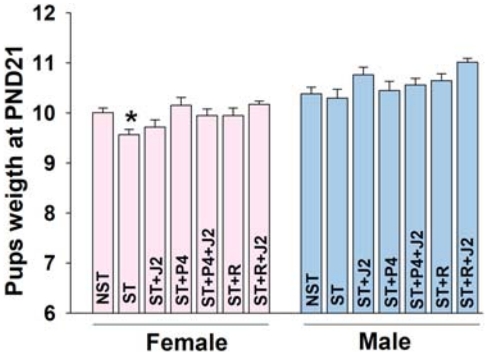

Figure EV2. Impact of prenatal stress and 15dPGJ2, alone or in combination with P4 or R5020 on the weight of female and male pups at postnatal day 21 (PND21).

Body weight were measured on PND21 for both female and male offspring mice from control (NST) or subjected to maternal restraint stress between E8-18 and received from E14 to 18 injection of placebo (ST), or +15dPGJ2 (ST + J2), or +P4 (ST + P4) or +P4 + 15dPGJ2 (ST + P4 + J2), or +R5020 (ST + R) or +R5020 + 15dPGJ2 (ST + R + PJ2). Bars represent mean ± s.e.m.; $^*P = 0.005$ vs. NST, analyzed by Mann–Whitney U test. Total number of female pups measured: NST $n = 44$, ST $n = 32$; J2 $n = 38$; P4 $n = 37$; J2 + P4 $n = 30$; R $n = 20$; and J2 + R $n = 23$; male pups measured: NST $n = 26$, ST $n = 27$; J2 $n = 43$; P4 $n = 33$; J2 + P4 $n = 32$; R $n = 27$; and J2 + R $n = 28$. Note that R5020 was only single injection given on E15.

© The Author(s)

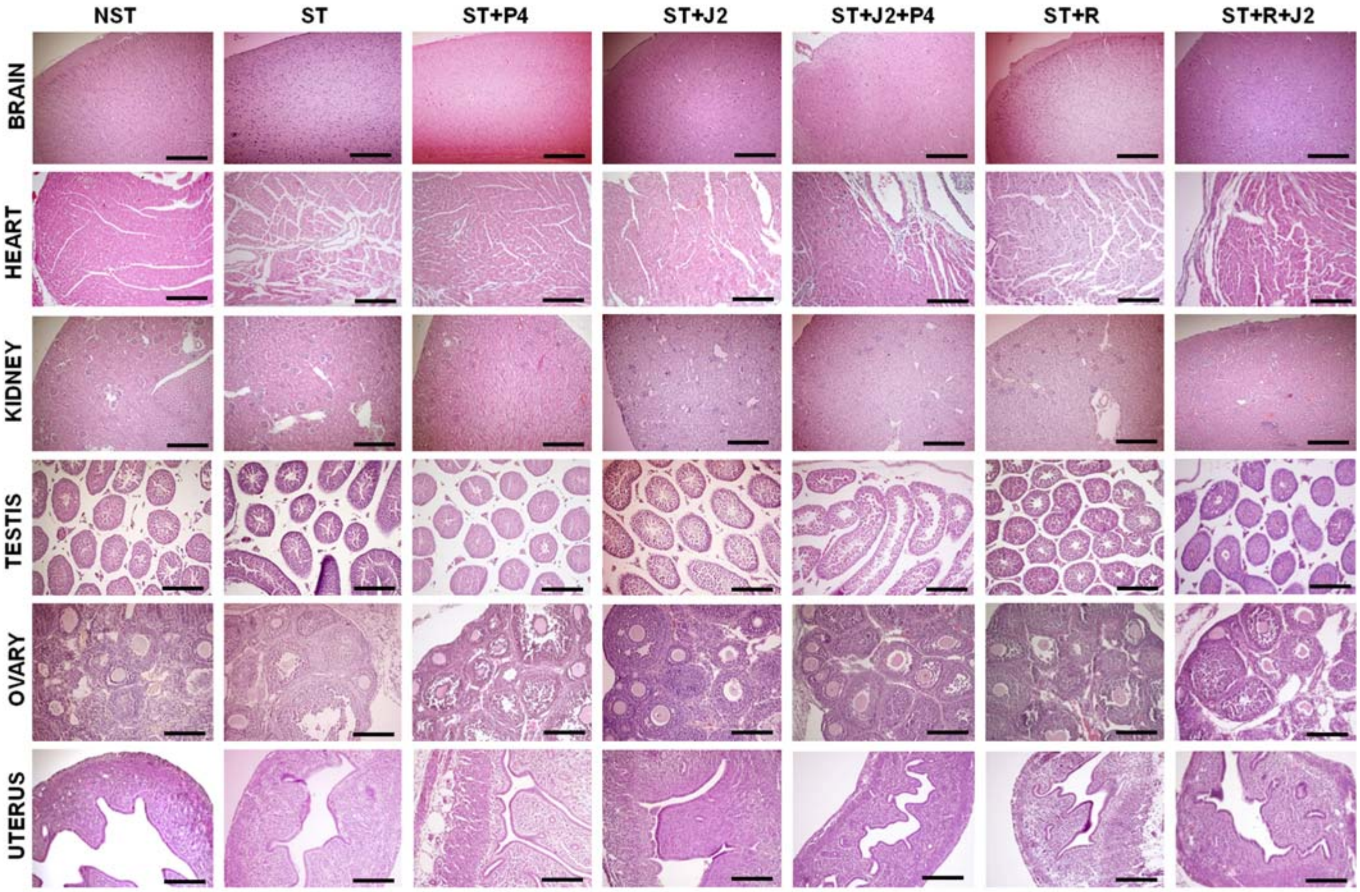

Figure EV3. Histologic comparison of the brain, heart, kidney, testis, ovary, and uterus on postnatal day 21 (PND21).

H + E stained tissues were compared for any signs of hemorrhage, fibrotic or necrotic areas and/or inflammatory leukocyte infiltration. Representative pictures of H + E stained tissues obtained from pups of time-pregnant wild-type mice left undisturbed state as control (NST) or subjected to maternal restraint stress and received injection of placebo (ST), or +15dPGJ2 (ST + J2), or +P4 (ST + P4) or +P4 + 15dPGJ2 (ST + P4 + J2), or +R5020 (ST + R) or +R5020 + 15dPGJ2 (ST + R + PJ2). *N* = 5 biological replicates. Scale bars = 300 µm.

 © The Author(s)
